# The diagnosis “failure to thrive” and its impact on the care of hospitalized older adults: a matched case-control study

**DOI:** 10.1186/s12877-020-1462-y

**Published:** 2020-02-14

**Authors:** Clara Tsui, Kristine Kim, Martha Spencer

**Affiliations:** 1 Department of Internal Medicine, University of British Columbia, 2775 Laurel St. Vancouver, Vancouver, BC V5Z 1M9 Canada; 2 Division of Geriatric Medicine, University of Ottawa, Ottawa, Canada; 3 Division of Geriatric Medicine, University of British Columbia, Vancouver, Canada

**Keywords:** Failure to thrive, Failure to cope, Acopia, Social admission, Ageism

## Abstract

**Background:**

“Failure to thrive” and associated diagnoses are non-specific terms applied to older adults when there is lack of diagnostic clarity and imply an absence of medical acuity. We investigated the effect of such admission diagnoses on delivery of patient care in a cohort of older adults admitted to a tertiary care teaching hospital.

**Methods:**

Retrospective matched cohort study conducted at a tertiary care hospital in Vancouver, BC. Cases identified were adults aged ≥65 years admitted to acute medical wards with an admission diagnosis of “failure to thrive”, “FTT”, “failure to cope”, or “FTC”, between January 1, 2016 and November 1, 2017 (*n* = 60, median age 80 years). Age-matched controls met the same inclusion criteria with admission diagnoses other than those of interest (*n* = 60, median age 79 years).

**Results:**

The primary outcome was time to admission, measured from time points in the emergency room that spanned from triage to completion of admission orders. Secondary outcomes were concordance of admission and discharge diagnoses and length of stay in hospital.

The total time from triage to admission for older adults admitted with FTT and associated diagnoses was 10 h 40 min, compared to 6 h 58 min for controls (*p* = .02). Concordance of admission and discharge diagnoses was only 12% for the “failure to thrive” cohort, and 95% for controls. Notably, 88% of the “failure to thrive” cohort had an acute medical diagnosis at the time of discharge. Patients in this cohort stayed 18.3 days in hospital compared to 10.2 days (*p =* .001).

**Conclusions:**

Patients with an admission diagnosis of FTT or other associated diagnoses had significant delays in care when presenting to the emergency room, despite often having acute medical conditions on presentation. The use of this non-specific label can lead to premature diagnostic closure and should be avoided in clinical practice.

## Background

“Failure to thrive”, or FTT, is a nonspecific term commonly applied to older adults in the emergency room when there is uncertainty over the cause of their presentation to hospital. This term was adopted from pediatrics in the 1970’s, and over the years, has come to represent a syndrome of vague symptoms among older adults that includes unexplained loss of appetite, weight loss, cognitive and functional decline, and social isolation, complicated by multiple medical comorbidities and psychiatric factors [1–4]. Despite the wide range of symptoms the term encompasses, and the lack of consensus in its definition, the term has been adopted into the International Classification of Diseases, Ninth Revision (ICD-9) since 1979 and continues to be commonly used in clinical practice [1].

The prevalence of FTT and related terms has not been quantified, however there has been a general trend of an increasing number of older adults presenting to healthcare, as their prevalence increases in the general population [5–7]. Concurrently, emergency department (ED) wait times, allocation of hospital resources, and the evolution of interdisciplinary care are at the forefront of healthcare discussions [8, 9]. Observational studies have shown that the emphasis on wait times and speed of patient flow as part of a “performance management approach” in resource allocation have shifted importance to efficiency rather than safe, patient-centered care [8]. It has been postulated that FTT and “failure to cope” are being used as labels of expediency to imply predominantly social issues, rather than medical issues, as the reason for admission [6, 10].

Studies have also suggested that labels are powerful in the healthcare setting and may alter how patients are perceived [6, 11]. An earlier retrospective cohort study hypothesized that acute medical illness rather than social factors is the primary reason for admission for patients labeled as FTT, as the majority of patients in this study received an extensive medical workup and interventions such as intravenous fluids and antibiotics [12].

To date, there have been no studies looking at potential harms that may occur when patients are admitted with a diagnosis of FTT and associated labels. The main objective of our study is to investigate the effect of such labeling on the admission process through the ED as a proxy for measuring delivery of care. We also sought to determine the concordance of the FTT admission diagnosis with the diagnosis at the time of discharge to determine if this label was maintained throughout the hospital admission. We hypothesized that this diagnostic label would be associated with delays in care and that many patients admitted with the FTT label would be later found to have acute medical diagnoses.

## Methods

### Study participants

**Fig. 1:**
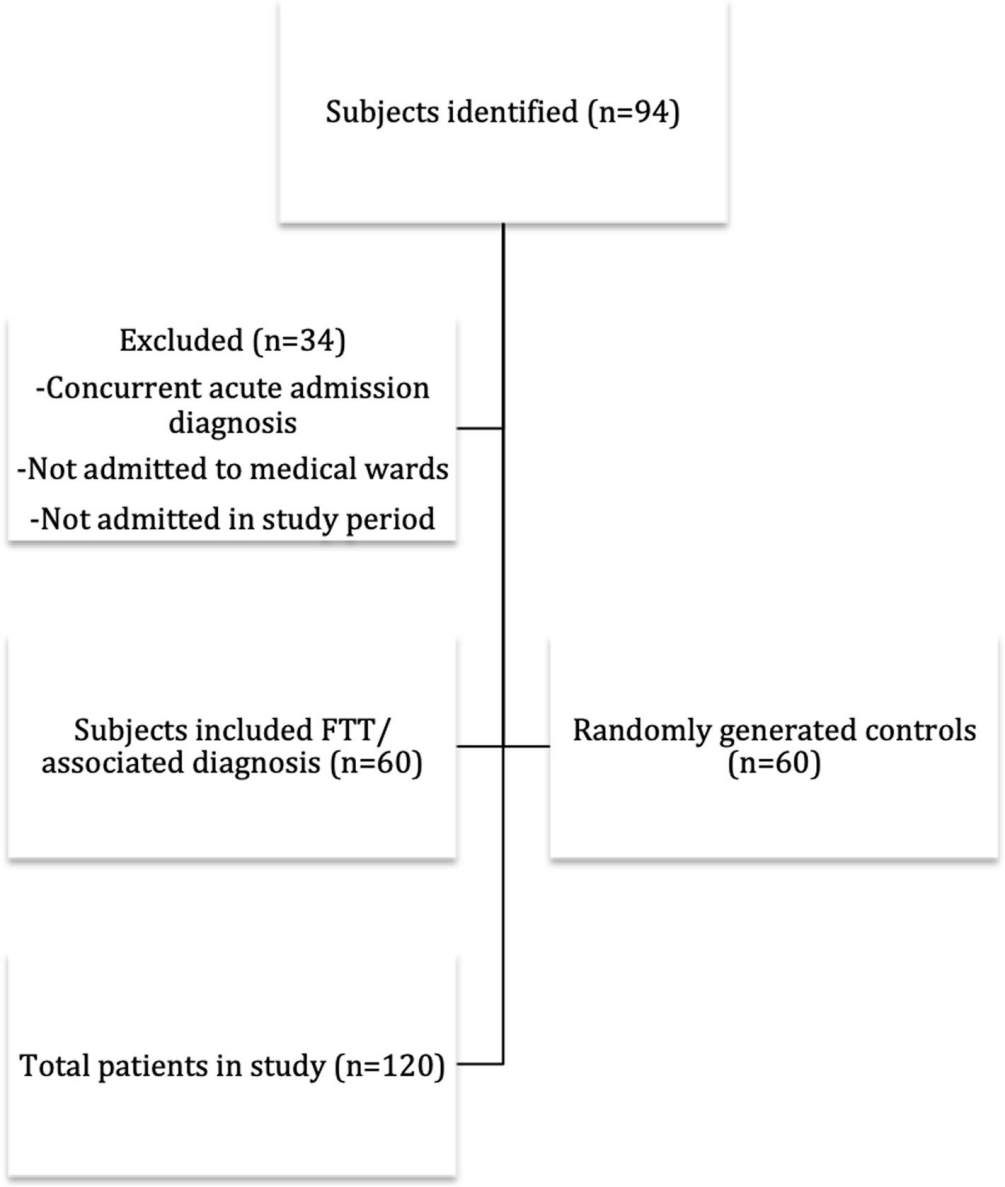
Process of case selection

Identified cases for the study were older adults aged ≥65 years who were admitted with diagnoses of failure to thrive, FTT, failure to cope, or FTC, between January 1, 2016- November 1, 2017 to medical wards under general internists or family physicians. Medical trainees (residents and medical students) worked on both services. The same inclusion criteria applied to randomly generated control cases, but who had admission diagnoses other than those of interest (Fig. 1).

### Ethics

We obtained approval from the University of British Columbia-Providence Health Care Research Ethics Board (UBC-PHC REB) prior to initiating data collection. Individual participant consent was deemed unnecessary for this minimal risk research study, according to Canadian regulations as laid out in Article 3.7 of the Tri-Council Policy Statement: Ethical Conduct for Research Involving Humans (TCPS2).

### Data collection

Patient charts were accessed via the hospital’s electronic medical record system. Basic demographic data collected included date of birth, age, and gender, as well as admission and discharge diagnoses and in-hospital mortality. Elements on past medical history included in the Charlson Comorbidity Index were also collected. Time at triage, time of referral to the admitting service and time of departure from the emergency room had timestamps that were electronically logged. Times of assessment by the emergency room physician and admission service resident were found on the emergency room discharge summary or the consultation sheet.

Since vital signs and other objective clinical findings at triage were not reliably or consistently recorded, investigations ordered and interventions performed in the ED were used as markers of patient acuity. These included basic bloodwork (complete blood count, electrolytes, glucose, and creatinine), intravenous antibiotics, blood cultures, chest x-ray, and computed tomography (CT) scans. Lastly, involvement of specialized geriatric care (geriatric emergency nurse, geriatric medicine) was noted.

While it is recognized that data pertaining to patients’ social situation (ex. living arrangement, social network), function (ex. activities of daily living, use of gait aid) and other markers of frailty (ex. nutrition, gait speed), such information was rarely documented and therefore not included.

### Statistical analysis

We calculated averages and medians for discrete variables and applied Student’s T-test to look for statistical significance, using a cutoff of *p* < .05.

## Results

### Patient population

A total of 120 patients were included in the study, half of whom had an admission diagnosis of FTT and the other half who had admission diagnosis other than FTT who were age-matched to the FTT sample. There were no significant differences in demographics or presence of comorbid conditions between the groups. These results are summarized in Table 1.

**Table 1:** Demographics, FTT group compared to controls

	FTT, n (%)	Controls, n (%)	*p-*value
Age (years)			
65–74	18 (30%)	19 (32%)	
75–84	25 (42%)	24 (40%)	
85+	17 (28%)	17 (28%)	
Mean ± SD	80 ± 9.1	80 ± 9.0	0.38
Gender			
Female	31 (52%)	28 (47%)	
Male	29 (48%)	32 (53%)	
Multimorbidity ^a^			
0–5	16 (26%)	20 (33%)	0.10
6–10	32 (53%)	32 (53%)	0.54
> 10	12 (20%)	8 (14%)	0.09
Mean ± SD	7.1 ± 1.2	6.8 ± 0.9	0.13

^a^Calculated using the Charlson Comorbidity Index

### Delays in care

In our cohort, patients admitted with FTT spent significantly more time in the ED, mainly from delays to physician assessment by both the emergency physician and the admitting service (Table 2). It was noted that there was a trend toward FTT patients remaining in the ED longer than the control group although this did not reach statistical significance. Notably, an abbreviated set of admission orders used at this institution to expedite the admission process was used 16 times in the control group, compared to only 3 times in the FTT group. The average length of stay in hospital for patients admitted with FTT was 18.3 days, compared to 10.2 days for the control group (*p =* .001).

**Table 2:** Mean times in admissions process and times spent in the emergency department

	FTT (hr:min)	Controls (hr:min)	*p-*value
Times from			
Triage to ERP	2:00	0:49	0.02*
ERP referral to admitting service	4:29	3:43	0.21
Referral to assessment by admitting service	4:13	2:41	0.04*
Total times			
Triage to admission	10:40	6:58	0.001*
Triage to ward	16:30	13:55	0.07

*Indicates statistical significance

### Medical acuity

In addition to delays in the admissions process and prolonged stay in the ED, there were significant discrepancies between admission and discharge diagnoses in the FTT group. Diagnoses were categorized as acute (ex. infections, falls, cardiac disease, drug side effects, or systems-specific issues, such as renal failure, and gastrointestinal bleeds), chronic (deconditioning, dementia and other progressive neurological disorders) and mixed (both acute and chronic conditions listed). In this cohort, 12% (*n* = 7) had FTT as a discharge diagnosis and the remaining 88% (*n* = 53) had acute medical diagnoses. In contrast, 95% (*n* = 57) of the control group had concordant discharge and admission diagnoses. Of the 5% (*n* = 3) in the control group who were discharged with other issues, 2 were “multifactorial falls”, and 1 was FTT). These are summarized in Table 3.

**Table 3:** Summary of admission and discharge diagnoses

	FTT, *n* (%)	Controls, *n* (%)
Chronicity		
Acute	53 (88%)	57 (95%)
Chronic	5 (9%)	2 (3%)
Mixed	2 (3%)	1 (2%)
Admit diagnosis		
FTT^a^	60 (100%)	–
Infection	–	22 (36%)
Cardiac	–	11 (18%)
GI	–	6 (20%)
Drug-related	–	2 (3%)
Fall	–	5 (8%)
Renal	–	4 (6%)
Neurologic	–	10 (16%)
Discharge diagnosis		
FTT^a^	4 (6%)	1 (2%)
Infection	21 (35%)	23 (38%)
Cardiac	14 (23%)	11 (18%)
GI	5 (8%)	5 (8%)
Drug-related	1 (2%)	2 (3%)
Fall	7 (12%)	5 (8%)
Renal	1 (2%)	3 (5%)
Neurologic	7 (12%)	10 (16%)

^a^And related terms: acopia, failure to cope or FTC

There were no significant differences between the frequency of investigation and use of intravenous antibiotics between the FTT and non-FTT groups. Additionally, there were no statistically significant differences in time to first blood draw, imaging performed (chest x-ray or CT scan), or administration of antibiotics (Table 4). Interestingly, double the number of patients in the non-FTT group did receive antibiotics, and had a higher number of blood cultures drawn.

**Table 4:** Frequency and time to first bloodwork, investigations, and intravenous antibiotics in FTT group compared to controls

	FTT		Controls (hr:min)		*p-*value
	hr:min	n (%)	hr:min	*n* (%)	
Bloodwork^a^	3:49	59 (98%)	2:10	60 (100%)	0.20
Antibiotics	1:19	15 (25%)	1:40	30 (50%)	0.24
Blood cultures	1:55	25 (42%)	0:48	42 (70%)	0.12
Chest X-ray	1:25	47 (78%)	0:47	50 (83%)	0.11
CT scans	2:20	34 (57%)	3:20	23 (38%)	0.35

^a^Bloodwork included basic complete blood count, electrolytes, glucose, and creatinine

### Other outcomes

In-hospital mortality was not significantly different between the two groups, at 10% (*n* = 6) for the FTT group compared to 8% (*n* = 5) for the controls. 38% (*n* = 23) of patients with FTT had Geriatrics involvement, compared to 8% (n = 5) for the controls. Geriatrics involvement was not associated with longer length of stay.

## Discussion

Several studies have shown that the term FTT is often applied to older adults in the acute medical setting with the implication that there is a social, rather than medical, reason for presentation to hospital [12, 13]. However, no study to date has studied the effect of this label on delivery of care. Our study suggests that many older adults with an admission diagnosis of FTT are in fact medically acute, and that there may be an association between this label and delays in care.

The medical acuity of this cohort of older adults is evidenced by two main findings. Firstly, of those admitted with FTT, 88% had acute medical diagnoses at time of discharge. Interestingly, this observation has been noted in an older study that found the related term “acopia” was recorded as a discharge diagnosis in only 12% of their cohort of 109 patients admitted with that label [14]. Another study identified that the most common diagnoses were malignancies and their associated sequelae, infections, and dehydration [3].

A second factor that points to medical acuity is that this cohort received a number of investigations and interventions in the ED including blood work, imaging and intravenous antibiotics. There was also no statistical difference between the frequency and timing of investigations and use of IV antibiotics between the FTT group and the controls (Table 4). That observation had also been previously reported, where 35% of patients admitted with FTT received IV antibiotics and 56% received CT scans (25 and 57% respectively in our cohort) [12]. Interestingly, double the number of non-FTT patients in our study received antibiotics and blood cultures compared to the FTT group while in the ED. This may suggest that patients labeled with an acute medical diagnosis at time of admission were more likely to be perceived as medically acute by ED staff, in contrast to patients labeled as FTT. Unfortunately, the scope of our data collection did not document whether patients in the FTT group went on to receive antibiotics and blood cultures later in their admission.

Our cohort of patients labeled FTT is medically acute, yet they experienced delays throughout their trajectory in the ED. Previous studies have shown that older adults with similar labels are medically active, yet these studies did not include objective measurements of delays in care, such as length of time to physician assessment, admission, and length of stay in the ED. [3, 12, 14]

Previous studies have identified several factors that may pose challenges in the management of older adults in the ED. These include the presence of atypical presentations, polypharmacy, multimorbidity, and barriers in communication stemming from sensory impairment, baseline cognitive impairment, and/or superimposed, evolving delirium [9, 15]. The shortage of resources and the emphasis on efficiency of patient flow through the ED further compounds these challenges. While assigning a label of FTT may be considered as a way to increase efficiency, our study shows that the use of this term on admission is associated with more prolonged trajectories through the ED and longer overall lengths of stay in hospital. In turn, this increases the risk of functional decline during and following hospitalization, with resulting loss of independence, higher risk of readmission, and increased mortality [3, 16]. Notably, Geriatrics involvement did not prolong lengths of stay in hospital in either group, which was included in our study to determine if subspecialty involvement could have contributed to the difference. Previous studies have shown that subspecialty involvement did prolong lengths of stay for older adults, but this was confounded by features that necessitated subspecialty care, like increased morbidity, cognitive impairment, and functional dependence [17].

With awareness of these factors unique to older adults, Acute Care of the Elderly units have become more abundant, yet few models exist to address the need for similar care models in the ED. One conceptual model proposed includes a frailty assessment at time of presentation, assignment of case managers to frail older adults, and creation of an intermediate care area to transition those patients out of the ED. [18] While such models have yet to be tested, logistical reworking and resource redistribution is only part of the solution, as the use of FTT and associated terms is also grounded in negative perceptions of older adults among practicing physicians and trainees [19–21].

The term FTT suggests an inherent “failure” on the part of the patient and is unfortunately often perceived as a part of normal aging. The term perpetuates the stereotype of older adults as “demented and decrepit”, and being more prone to “aches and pains”, “mental slowness” and “worrying more” [13, 19]. As a result, complaints like pain, fatigue, depression, and worsening cognition could be wrongfully attributed to a patient’s age, missing critical clinical cues for an underlying, undiagnosed condition [19]. Systematic reviews and qualitative studies found that medical students preferred younger patients with acute diseases that can be “cured”, as opposed to older adults who required more “care”, as they tended to have a number of medical problems and atypical disease presentations that required more time to elucidate [20, 21].

Older adults labeled as FTT are medically acute and therefore require urgent care. Our study suggests that there may be an association between this term and delays in care, which supports conclusions from previous studies that the use of the label may “hinder the urgent search for treatable, reversible causes of deterioration” [13]. Therefore, the use of the label FTT is problematic and potentially harmful to older adults presenting to acute care. We suggest instead using the symptoms described by the patient as a working admission diagnosis, such as “weakness” or “dyspnea” for which there is a differential diagnosis. Other alternatives in the absence of medical descriptors could be considered, such as “decline in function”, “cognitive decline”, or even “frailty”. Importantly, those terms also have ICD codes and are therefore accepted diagnoses on medical documentation.

### Study limitations

While this study captured a relatively sizeable cohort of FTT patients compared to other studies on this topic, it was performed at a single, urban academic institution, which may limit generalizability. We also acknowledge that our findings may only pertain to medicine patients, as no surgical patients were included in our study population. Due to the case-control design of our study, we also recognize that our results represent an association between FTT and delays in care and not causation.

We were not able to determine which health care practitioners initially assigned this label, due in part to lack of written communication between emergency room physicians and admitting services. Even with documentation, there was a noted lack of explanation as to why this term was used. As such, the timing of when this diagnosis was applied is uncertain, and qualitative studies are underway to better ascertain when and where the term first originates. We have also used the Charlson Comorbidity Index as a way of accounting for differences in medical complexity between the control and FTT cohorts but would have preferred to calculate frailty scores should that information have been available to us on the electronic medical record. Valuable information such as functional, nutritional, and cognitive status, medications, or comprehensive geriatric assessments were not reliably available in all patient charts, so these components were not included in the study. Subsequent follow-up after discharge from hospital, such as re-admission or mortality, were unfortunately not included in the study due to the limitations of a chart review design.

### Future directions

This study has formed the basis for a qualitative study in which health care practitioners will be interviewed to explore why the term FTT is used in our health care system. It is anticipated that the information obtained during this qualitative study will inform education and interventions to reduce the use of this label for older adults presenting to acute care.

## Conclusion

Although previous studies have shown that older adults with diagnosis of FTT are admitted for acute illnesses rather than social factors, this is the first study to show an association between an admission diagnosis of FTT and delays in care. The use of this term provides little clinically useful information, and may ultimately pose harm to older adults by delaying diagnosis and delivery of appropriate care. As such, it is our opinion that the use of FTT and associated terms should no longer be used in clinical practice.

## Data Availability

The datasets used and/or analyzed during the current study are available from the corresponding author on reasonable request.

## Abbreviations

ED: Emergency department
FTC: Failure to cope
FTT: Failure to thrive
ICD: International Classification of Diseases
